# The Application of Earth in Surgery

**Published:** 1873-10

**Authors:** 


### The Application of Earth in Surgery.

It was my good fortune during the winter to be residing in the same hotels, both at Nice and Rome, with the eminent American surgeon, Dr. Hewson, of Philadelphia, who brought to my notice the advantages attending the use of earth as a dressing in surgical cases. He told me he attributed his great success at the Pennsyl-

*Physioligie, 2 Auflage, 1872, p. 634.

vania General Hospital—a success which has been remarkable since the American war, especially in connection with cases of amputation—entirely to the use of this agent. The results of its use which have come more or less directly under my own observation have been so successful that I venture to send you brief outlines of the cases.

1. A case of epithelioma of the cheek, of twelve years' standing. In December there was an ulcer on the right cheek larger than a half-crown piece, which was being treated with dry-earth applications. In March I saw the case dressed in Rome, as did also Dr. Pantaleoni and other physicians. There were then, instead of the extensive ulcer of three months before, superficial points of ulceration which, had they been collected together, a fourpenny-piece would have covered. The patient, a gentleman of sixty-two years of age, was not very sanguine, having had the unfavorable opinions of several distinguished surgeons, but he expressed himself most gratefully for the relief from pain and from the horribly fetid odor, which made life a burden to him before the earth was applied.

2. A physician at Mentone, under Dr. Hughes Bennett's care I believe, sent for Dr. Hewson from Nice to resect his knee-joint, or to amputate his leg. Dr. Hewson found an abcess, thought to be metastatic, in the head of the fibula. He begged to be allowed to try earth dressing before proceeding to an operation. The case did perfectly well, and the joint is apparently as good as ever.

3. The American chaplain in Rome was thrown from his horse, and struck his leg against a rail. The trowsers were not cut, but the tibia, the spine of which was laid bare for several inches, sustained a comminuted fracture, and the tibialis anticus was partly stripped from its attachment. Dr. Hewson put in two or three stitches, and applied earth dressing. The patient had scarcely any pain; there was but little discharge, and the leg rapidly healed. The almost total absence of pain was very remarkable.

4. At Naples a poor woman accosted me in the street, and asked me to cure a large chronic ulcer on the right leg, which extended from the external malleolus to midway between the ankle and knee. I offered her a few soldi, and told her to go to the hospital. She said she was not begging for money, but to be cured, and as I was an English doctor, I could cure her if I would. Her grandfather had been in the service of an English nobleman, it seemed. I managed to find some light-yellow clay, allowed it to dry, pounded it and sifted it through fine muslin, and applied it to the sore, covering it with ordinary grocer's blue paper, the edges of which I wetted in order to make it adhere to the skin. I told the poor creature to remain at home and rest the ulcerated leg on a chair even when she sat spinning at her door. The next morning I found her waiting for me at the gate of the hotel. She said she was quite free from pain; she said she slept all night, a thing she had not done for months before; and with tears in her eyes she thanked me for the relief I had given her. In three weeks the ulcer was almost well. It is right to say I took means to improve the general health.

5. An American gentleman in Rome asked me to prescribe for him, as he had gonorrhœa. The gonorrhœa was very acute, with chordæ and severe pain during micturition. As he bound me to secrecy, I could not borrow a small double-tubed catheter which Dr. Hewson uses in these cases, and was obliged to inject a large quantity of muddy water with an ordinary syringe. The patient was greatly relieved by the first application, which was repeated on the two succeeding days, though the discharge and pain had ceased on the second day. The patient had a dose of chloral and an aperient each night.

6. A young Roman, a friend of the previous patient, came to me complaining of rheumatism. He had had a gleet eight months. I injected the urethra with muddy water every other morning for ten days, when the discharge ceased. The rheumatic pains ceased also, supporting, therefore, my friend Dr. Bond's theory of the cause of gonorrhœal rheumatism. This patient took iron and arsenic in small doses, as he was somewhat anæmic.

The freedom from pain, the destruction of all odor from the wound, the diminution of the discharges, and the rapid healing after the application of the earth, were the features common to all these cases which impressed me most.

Dr. Hewson, who will shortly visit England, believes the action of the earth mainly a chemical one, and he covers his dressing with blue paper in order that the chemical rays of light may be admited to the wound.—*Joseph Groves, B. A., in London Lancet.*

